# Chemical Exposures: The Ugly Side of Beauty Products

**DOI:** 10.1289/ehp.113-a24

**Published:** 2005-01

**Authors:** Julia R. Barrett

In recent decades reproductive and developmental problems have become more prevalent—for example, data from the Centers for Disease Control and Prevention (CDC) show that male reproductive problems, including undescended testicles and hypospadias, doubled between 1970 and 1993. Environmental chemicals are strongly suspected to be contributing factors. Several recent reports highlight the presence of low-level concentrations of potential reproductive or developmental toxicants, particularly phthalates, in cosmetics and personal care products. A key question is whether these exposures are significant enough to cause harm.

In June 2004, Environment California issued *Growing Up Toxic: Chemical Exposures and Increases in Developmental Diseases*, which details chemicals found in consumer products and their potential health impacts. Other reports released around the same time by the Environmental Working Group (*Skin Deep: A Safety Assessment of Ingredients in Personal Care Products*) and Friends of the Earth (*Shop Till You Drop? Survey of High Street Retailers on Risky Chemicals in Products 2003–2004*) support Environment California’s publication.

According to these three reports, makeup, shampoo, skin lotion, nail polish, and other personal care products contain chemical ingredients that lack safety data. Moreover, some of these chemicals have been linked in animal studies to male genital birth defects, decreased sperm counts, and altered pregnancy outcomes. There is no definitive evidence for the same effects in humans, but widespread exposure, primarily to phthalates, has been shown to occur.

Phthalates, as key components in plastics, appear in many consumer products. The main phthalates in cosmetics and personal care products are dibutyl phthalate in nail polish, diethyl phthalate in perfumes and lotions, and dimethyl phthalate in hair spray. Often, their presence is not noted on labels.

“The concerns that are focused around this particular chemical [class] have arisen from a series of tests and studies that have been released recently that point to significant potential health concerns,” says Sujatha Jahagirdar, an environmental advocate with Environment California. For example, a population study conducted by the CDC and published in the March 2004 issue of *EHP* demonstrated that 97% of 2,540 individuals tested had been exposed to one or more phthalates. Another preliminary study conducted at the Harvard School of Public Health and published in the July 2003 issue of *EHP* showed a correlation between urinary phthalate metabolite concentrations and DNA damage in human sperm. However, exposure sources in this study were unknown.

The personal care industry remains confident about phthalate safety, however. The Cosmetic Ingredient Review panel, an independent research group sponsored by the Cosmetic, Toiletry, and Fragrance Association, published a detailed literature review in February 2003 that unequivocally states that current use of phthalates in cosmetics and personal care products is safe. Marian Stanley, manager of the Phthalate Esters Panel of the American Chemistry Council, says, “Some of these concerns [from environmental groups] are based on high-dose animal testing. The exposure that we really see in people—and we have the CDC numbers to back that up—is remarkably low. To us, why bother getting rid of a highly useful product when there should be no concern?”

Therein lies the controversy—environmental groups view the CDC data as evidence of widespread exposure, whereas industry groups view it as evidence of low-level exposure that falls well below amounts shown to cause problems in animal studies. The environmental groups respond that although it may be low-level exposure, it is *chronic* low-level exposure. Says Elizabeth Sword, executive director of the nonprofit Children’s Health Environmental Coalition: “In my view there is sufficient evidence to pique my concern, not only as a parent but as the executive director of this organization, to circulate this information directly to parents in a way that they can then make the healthiest decisions.”

However, consumers cannot make such judgments without knowing the ingredients contained in the products they use. “There are industry trade secrets and formulations that for industry reasons are kept from the consumer,” says Sword. “This prevents the consumer from making fully informed decisions.”

Environment California and the other environmental organizations hope to change that through consumer education and policy reform at the state and federal levels. “Environment California is pushing for a commonsense chemical policy that requires chemical manufacturers to test . . . their chemicals before they are released into the market and also provide the public with the tools that it needs to protect itself from potential dangerous impacts,” says Jahagirdar. “Labeling is an extremely important and ethical thing for manufacturers to be doing.”

“I think a lot of this comes down to an individual’s acceptance of risk,” says Sword. “[Each person’s] personal risk tolerance is different. I think what we as a society need to feel confident about is that adults will at least make better decisions if you give them a way to do so, particularly when the health of a child may be at risk from making a bad decision.”

## Figures and Tables

**Figure:**
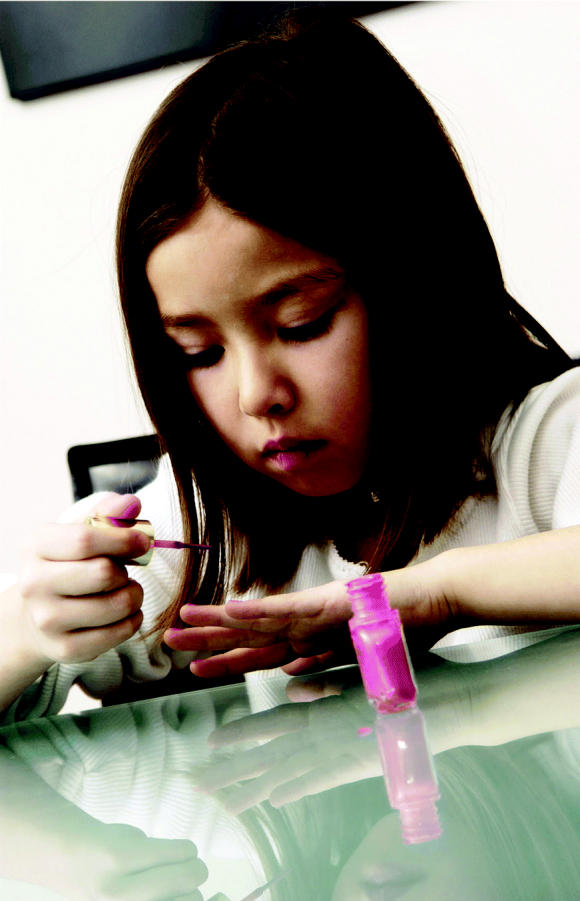
**Starting too young?** Concern is mounting over the effects of long-term exposures to chemicals—such as phthalates—found in cosmetics and personal care products.

